# Introducing a new *SLEEP Advances* initiative: Living Legends

**DOI:** 10.1093/sleepadvances/zpac039

**Published:** 2022-10-20

**Authors:** Mary A Carskadon

**Affiliations:** Department of Psychiatry and Human Behavior, Alpert Medical School of Brown University, Bradley Hospital Sleep Lab, Providence, RI, USA

The Sleep Research Society is 60 years old, the American Academy of Sleep Medicine is preparing to celebrate its fiftieth year, and other sleep societies around the globe are also maturing! Our field has achieved a remarkable record of discoveries, yet these accomplishments come with harsh news as the lives of our pioneering members pass on. I have felt the pain deeply in recent months and years with the recurrent losses of such giants as Michel Jouvet, Christian Guilleminault, William Dement, Rosalind Cartwright, Allan Rechtschaffen, Barbara Jones, Irwin Feinberg. Our field’s recent losses are too numerous for me to list here; however, my personal distress has galvanized me to open an initiative at *SLEEP Advances* to honor our living pioneers and to gain from them anew by inviting papers from those with early publications and sustained contributions in our field. I welcome Drs. Sonia Ancoli-Israel and Ralph Lydic as associate editors for this initiative and two SRS trainee members, Dr. Renske Lok and Katrina Rodheim, as editorial interns to assist authors and editors as needed.

I am delighted to announce our first Living Legends publication, “Looking back at a life in sleep research—and some thoughts for the future”, by Wallace Mendelson, MD. A second manuscript from David Kupfer, MD, with Ellen Frank, PhD, “David J Kupfer: Long day’s journey into sleep” will follow soon, with others on their way. I have so enjoyed reading and learning from these contributions; I hope that all SRS members—especially trainees—will enjoy them, too.

Mary A. Carskadon, PhD

*SLEEP Advances* Editor in Chief

15 October 2022

